# The Story of the Insane from Year to Year

**Published:** 1891-11-21

**Authors:** 


### The Story of the Insane from Year to Year.

(Fourth Series.)

### NORTHAMPTON COUNTY ASYLUM.

The patients in this asylum increased during the year from 713 to 753, showing an increase of 40; but it would seem that 12 only of these were from Northamptonshire. The admissions were 235; the recoveries 58, and the deaths 101. The percentages would be: Recoveries, 30·85; deaths, 13·4. Of the admissions 49 were cases of dementia, 17 were idiots, 6 were paralytics, and 19 were epileptics. Drink was the assigned cause in 12 instances, and hereditary influence in 63. Fifty-four of the deaths were caused by brain diseases, and 12 by phthisis. When speaking of the admissions Mr. Greene says, "Nothing that can be said by medical superintendents seems to have much effect in inducing the friends of patients to place them under treatment during the first stage of disease, although it is well known that its curableness is much greater than at any future stage. So, too, with the causes of insanity. It is certain that heredity is more common than any other cause whatever, and yet those having the taint marry and are given in marriage, apparently without a thought of the probable consequences." The asylum passed through an epidemic of scarlet fever, at least we are told the disease broke out in the children's block, and 14 patients and six nurses were stricken. Lectures on nursing are given to the staff by the medical officers. Sixteen of the nurses obtained certificates from the St. John's Ambulance Association. The farm accounts show a balance of £48 against the farm; but we notice that rent of land, patients' labour, and interest of capital are charged. The weekly cost of maintenance was 7s. 8⅜d.

### THE DORSET COUNTY ASYLUMS.

There were 447 patients in the asylum at the end of the year, being 14 more than at the close of 1889. There were 86 admissions; 29 recoveries, and 42 deaths. The recoveries were 33·7 per cent. on the admissions, and the deaths 9·1 on the average number resident. Excluding transfers the percentage of recoveries would rise to 36·2. Of the year's admissions ten were driven insane by drink, and twenty-four by hereditary tendency. Of the deaths fourteen were due to phthisis.

When speaking of the recent proposal to establish special hospitals for the treatment of insanity, Dr. Macdonald says, "The construction and maintenance of special hospitals would of necessity entail an enormous expenditure, and whatever development the future may witness, the present tendency is to mould existing asylums to suit all purposes."

The Lunacy Act is referred to as follows: "The Lunacy Act of 1890 has had a disastrous effect on scientific work in asylums. We should not raise a discordant note, were we conscious of any advantage commensurate with the work the provisions of the Act entail. But knowing as we do that the Lunacy Commissioners were opposed to all these extra and laborious duties being thrust upon us, we smart under the injustice of being compelled by Act of Parliament to supply apparently useless returns and reports to fill the pigeon holes at Whitehall."

The Visiting Committee record their full approval of the management of the two asylums by the Medical Superintendent.

The balance in favour of the farm is given as £1,312. The sum of £19 10s. is put down as rent of land; but we see nothing for interest of capital invested, nothing for patients' labour, and the prices of articles supplied are not given. The average weekly cost per head is 8s. 5½d.

